# Supplementary material for: A two-phase core-plasma model for microvascular blood flow: Comparative analysis of hemodynamic models
Source: PLoS One. 2026 Jan 2;21(1):e0327948. doi: 10.1371/journal.pone.0327948 (PMC12758828; doi:10.1371/journal.pone.0327948)
Supplement: S1 Fig — Pressure drop (ΔP) is plotted against flow rate (Q) for 50 μm (top) and 25 μm (bottom) microchannels using PBS and plasma suspensions. Imposed and corrected ΔP values are compared to validate pressure bounds and confirm that upstream tubing losses are negligible. (PDF) [file pone.0327948.s001.pdf]

S1. Pressure drop analysis

Pressure drop and flow rate (ΔP–Q) plots for 25 and 50 µm round microchannels with PBS and plasma (Ht 5–20%). Black triangles are imposed inlet pressures from the controller (20–200 mbar); colored circles are microchannel-only pressures corrected for tubing losses, computed as $\Delta P_{\text{corr}}(Q) = \Delta P_{\text{meas}}(Q) - \Delta P_{\text{tube}}(Q)$ after measuring ΔP_{tube} by flowing water through the tubing without the chip. The near-linear trends confirm laminar (Poiseuille) flow. This figure validates that the imposed inlet pressure from the Fluigent controller accurately represents the pressure drop across the microchannel, confirming negligible upstream losses.

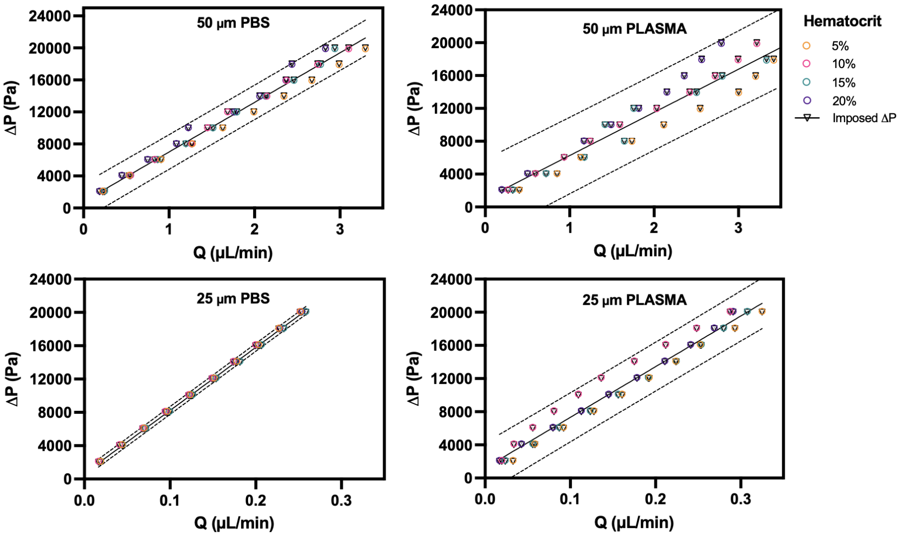

Fig S1.1. Pressure drop vs. flow rate in PBS and plasma suspensions. ΔP is plotted against Q for round microchannels of 50 µm (top) and 25 µm (bottom) diameter with PBS (left) and plasma (right). Black triangles denote the **imposed** pressure from the pressure controller (20–200 mbar). Colored circles show the **corrected** chip pressure for hematocrits 5–20%, computed by subtracting tubing losses measured separately (water, chip removed). Dashed lines are guides indicating the imposed-pressure bounds.
