# Supplementary material for: A two-phase core-plasma model for microvascular blood flow: Comparative analysis of hemodynamic models
Source: PLoS One. 2026 Jan 2;21(1):e0327948. doi: 10.1371/journal.pone.0327948 (PMC12758828; doi:10.1371/journal.pone.0327948)
Supplement: S2 Text — Description of Root Mean Square (RMS) error, one-way ANOVA, and Sidak multiple-comparison tests used to evaluate model fits and statistical significance. (PDF) [file pone.0327948.s002.pdf]

S2. Statistical analysis methods

Statistical analyses and data visualization were performed using GraphPad Prism (GraphPad Software, San Diego, CA, USA). The analyses included Root Mean Square (RMS) error calculations, ordinary one-way analysis of variance (ANOVA), simple linear regression, and significance testing with a confidence level of 95%.

Root Mean Square (RMS) Error: The Root Mean Square (RMS) error quantifies the deviation between experimental data and model predictions, providing a measure of model accuracy. It is calculated as:

$$\text{RMS Error} = \sqrt{\frac{1}{N}\sum_{i=1}^{N}(y_i^{\text{exp}} - y_i^{\text{model}})^2} \tag{1}$$

where y_i^{exp} represents the experimentally measured values, y_i^{model} are the corresponding theoretical predictions, and N is the total number of data points. A lower RMS error indicates a better fit between the model and experimental data, while a higher RMS error suggests greater discrepancies.

Ordinary One-Way ANOVA with Sidak Multiple Comparisons: Ordinary one-way Analysis of Variance (ANOVA) is used to determine whether there were statistically significant differences between multiple experimental groups. ANOVA tests the null hypothesis that all group means are equal by comparing the variance within groups to the variance between groups, yielding a p-value that indicates the probability of observing the data if the null hypothesis were true. If a significant difference was detected ($p < 0.05$), Sidak's multiple comparison test was applied as a post hoc analysis to identify which specific groups differed from each other.

The significance levels are denoted as:

- **$p < 0.05$** (*) – Significant difference
- **$p < 0.01$** (**) – Strong significant difference
- **$p < 0.001$** (***) – Highly significant difference
- **$p < 0.0001$** (****) – Extremely significant difference
