# Supplementary material for: A two-phase core-plasma model for microvascular blood flow: Comparative analysis of hemodynamic models
Source: PLoS One. 2026 Jan 2;21(1):e0327948. doi: 10.1371/journal.pone.0327948 (PMC12758828; doi:10.1371/journal.pone.0327948)
Supplement: S3 Table — Fitted parameters for the Core–Plasma model showing flow behavior and consistency indices across applied pressures, hematocrit levels, and suspending media (PBS or plasma). (PDF) [file pone.0327948.s003.pdf]

S3. Core-Plasma model parameters n_{cp} and K_{cp} across different pressures, hematocrit levels, and suspensions

This section presents the fitted Core-Plasma model parameters n_{cp} (flow behavior index) and K_{cp} (consistency index) for RBC suspensions under varying inlet pressures, hematocrit levels, and suspending media (PBS or plasma). These values are obtained by fitting experimental velocity profiles to the Core-Plasma model, enabling quantitative comparisons of non-Newtonian behavior across conditions (Table S3.1).

Table S3.1. Core-Plasma model parameters n_{cp} and K_{cp} across different pressures, hematocrit levels, and suspensions.

**Core-Plasma n_{cp}**								
**Pressure (mbar)**	5% PBS	10% PBS	15% PBS	20% PBS	5% Plasma	10% Plasma	15% Plasma	20% Plasma
20	1.3661	1.4800	1.1921	1.3244	0.9246	0.8180	0.7786	1.0993
40	0.7776	1.0433	1.3113	0.5884	0.9641	0.6856	1.4997	0.9147
60	1.2152	1.2037	1.0603	1.3561	0.8416	2.0940	0.8258	0.7635
80	0.7764	0.8938	1.1545	0.8888	1.1717	0.9327	0.6000	0.7884
100	1.4460	0.9056	1.1428	1.0956	1.2265	0.8387	1.0244	0.8120
120	0.9208	0.9396	0.8065	1.8794	1.2200	0.4465	0.7574	0.8753
140	0.8604	1.3014	1.0240	1.5176	1.1433	1.1461	0.7343	1.0594
160	0.6735	2.5798	1.0117	1.0029	1.2331	0.9612	0.8489	0.9910
180	0.8531	1.1853	1.1308	1.0787	1.2071	1.0077	0.7479	1.0866
200	1.3496	1.8169	1.2590	1.4137	1.3408	0.8731	0.9712	0.7837

**Core-Plasma K_{cp} (Pa·s$^{n_{cp}}$)**								
**Pressure (mbar)**	5% PBS	10% PBS	15% PBS	20% PBS	5% Plasma	10% Plasma	15% Plasma	20% Plasma
20	7.27E-4	6.02E-4	1.25E-3	1.85E-3	1.08E-3	9.89E-3	3.75E-3	3.02E-3
40	5.54E-3	2.78E-3	7.09E-4	3.01E-2	1.03E-3	6.78E-3	6.78E-5	1.99E-3
60	7.36E-4	1.02E-3	1.31E-3	5.04E-4	2.21E-3	2.24E-6	3.55E-3	7.81E-3
80	6.13E-3	5.68E-3	6.97E-4	6.74E-3	2.88E-4	6.44E-3	1.31E-2	5.74E-3
100	9.82E-5	4.47E-3	7.18E-4	1.94E-3	2.00E-4	4.80E-3	8.88E-4	7.45E-3
120	3.05E-3	3.43E-3	6.10E-3	1.52E-5	1.82E-4	1.24E-1	3.80E-3	4.16E-3
140	4.68E-3	3.94E-4	2.33E-3	1.23E-4	2.78E-4	7.85E-4	5.69E-3	1.40E-3
160	1.29E-2	6.15E-8	1.79E-3	2.27E-3	1.57E-4	3.72E-3	3.43E-3	1.54E-3
180	8.68E-3	6.54E-4	8.42E-4	2.14E-3	1.84E-4	2.52E-3	7.00E-3	1.10E-3
200	3.24E-4	7.07E-6	3.78E-4	1.96E-4	6.82E-5	9.88E-3	1.65E-3	7.19E-3
