# Supplementary material for: A two-phase core-plasma model for microvascular blood flow: Comparative analysis of hemodynamic models
Source: PLoS One. 2026 Jan 2;21(1):e0327948. doi: 10.1371/journal.pone.0327948 (PMC12758828; doi:10.1371/journal.pone.0327948)
Supplement: S4 Figs — Supplementary figures summarizing model-derived viscosity and rheological parameters from the Carreau, Power Law, Core–Plasma, and Double-Parameter Power (DPP) models across hematocrit levels, suspensions, and channel diameters. (PDF) [file pone.0327948.s004.pdf]

# S4. Characterization of non-Newtonian and fitting parameters

This section presents supplementary figures related to the characterization of blood flow behavior in microfluidic channels. Model-derived parameters are shown across varying hematocrit levels, suspending media (PBS and plasma), and channel diameters (25 µm and 50 µm). Analyses include curve fitting using three non-Newtonian rheological models—Carreau (Fig. S4.1, Fig. S4.2), Power Law (Fig. S4.3), and Core-Plasma (Fig. S4.4)—as well as the Double-Parameter Power (DPP) Fit (Fig. S4.5), which is used to capture geometric features of the velocity profile (core and wall bluntness).

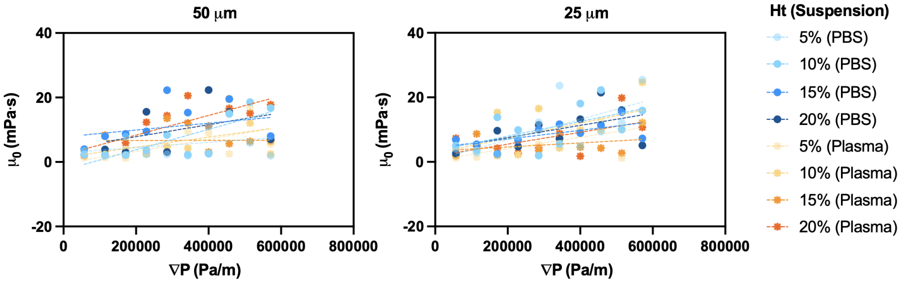


**Fig S4.1. Zero-shear viscosity trends from the Carreau Model across conditions.** Zero-shear viscosity ($\mu_0$) derived from the Carreau Model is shown for varying hematocrit levels and suspensions (PBS and plasma) in 50 µm (left) and 25 µm (right) microchannels. Data are plotted as a function of pressure gradient ($\nabla P$) and fitted using simple linear regression to visualize potential trends.

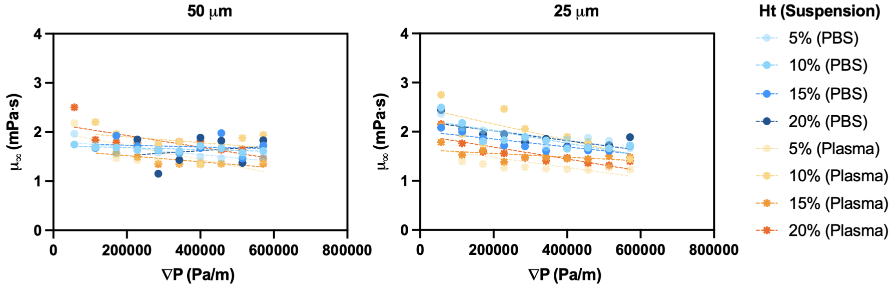


**Fig S4.2. Infinite-shear viscosity trends from the Carreau Model across conditions.** Infinite-shear viscosity ($\mu_\infty$) predicted by the Carreau Model is shown across varying hematocrit levels and suspension types (PBS and plasma) for 50 µm (left) and 25 µm (right) microchannels. Values are plotted against the pressure gradient ($\nabla P$) and fitted using simple linear regression to assess shear-dependent trends.

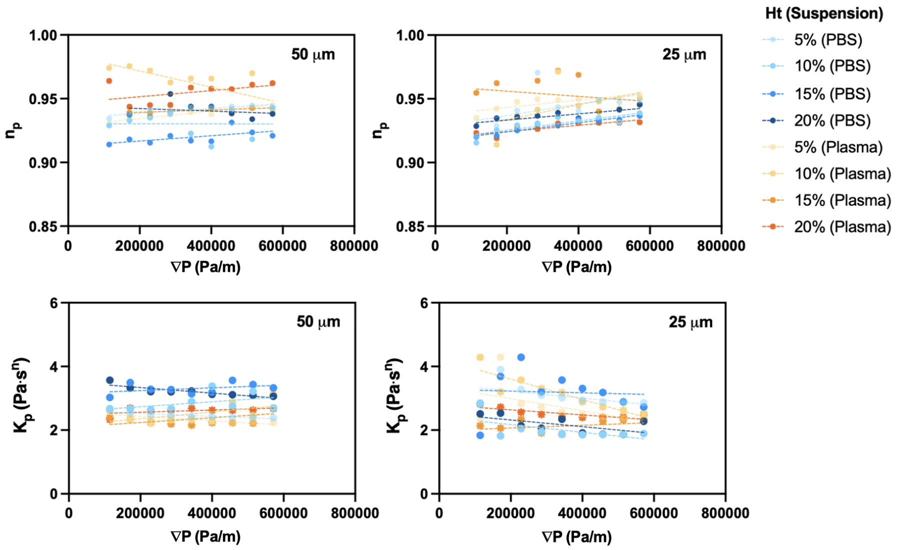


**Fig S4.3. Power Law Model parameters across varying hematocrit levels and channel sizes.** Power Law model parameters are shown for different hematocrit levels, suspensions (PBS and plasma), and channel sizes. (Top panel) Flow behavior index ($n_p$) is plotted as a function of pressure gradient ($\nabla P$) for 50 µm and 25 µm microchannels. (Bottom panel) Consistency index ($K_p$) is shown for the same conditions. Simple linear regression is applied to visualize parameter trends across varying flow and confinement conditions.

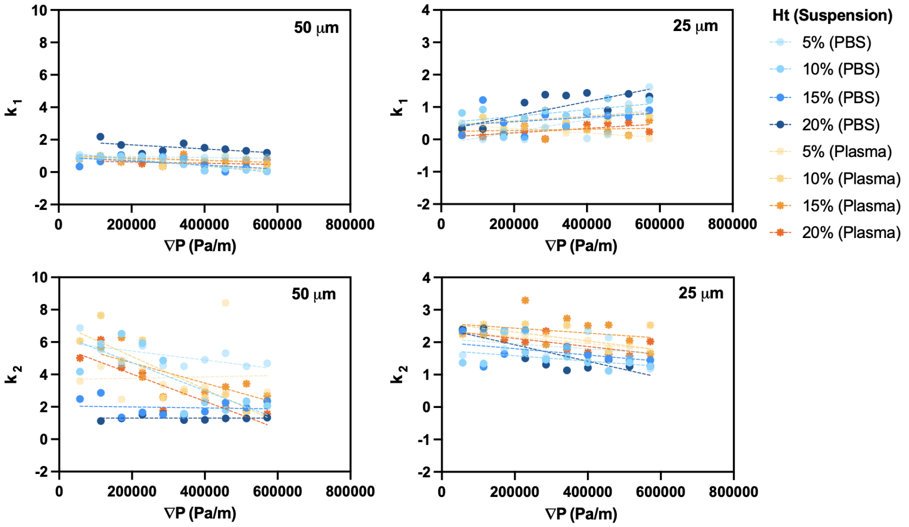


**Fig S4.4. Double-Parameter Power Fit parameters across varying conditions.** Variation of the Double-Parameter Power (DPP) Fit parameters—$k_1$ (core bluntness) and $k_2$ (wall bluntness)—is shown as a function of pressure gradient ($\nabla P$) for red blood cell (RBC) suspensions in PBS and plasma. Simple linear regression is applied to visualize trends across varying hematocrit levels and channel conditions.

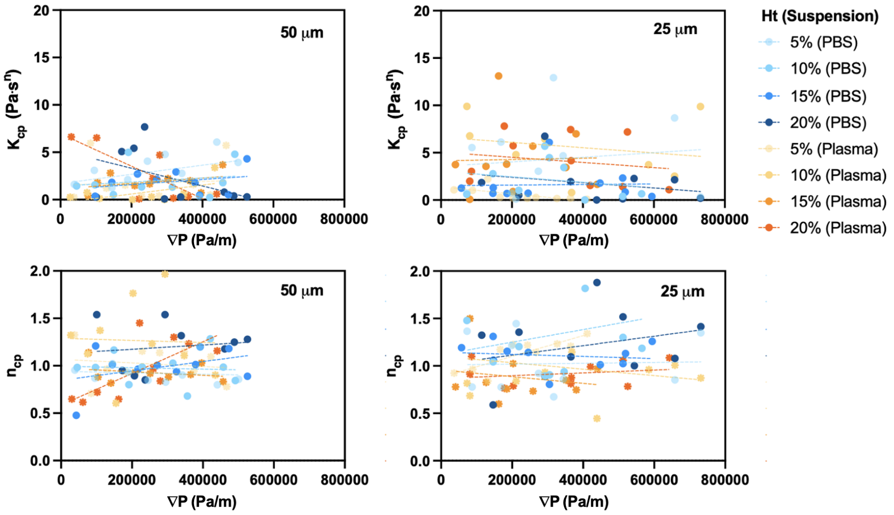


**Fig S4.5. Core-Plasma Model parameters across varying conditions.** Trends for the Core-Plasma Model parameters—consistency index ($K_{cp}$) and flow behavior index ($n_{cp}$)—are shown as a function of the pressure gradient ($\nabla P$). (Top panel) $K_{cp}$ values for 50 µm and 25 µm channels. (Bottom panel) $n_{cp}$ values for the same conditions. Simple linear regression is applied to assess parameter variability, with most cases showing no statistically significant trends.
