# Supplementary material for: A two-phase core-plasma model for microvascular blood flow: Comparative analysis of hemodynamic models
Source: PLoS One. 2026 Jan 2;21(1):e0327948. doi: 10.1371/journal.pone.0327948 (PMC12758828; doi:10.1371/journal.pone.0327948)
Supplement: S5 File — Measured volumetric flow rates and corresponding Reynolds numbers for 25 μm and 50 μm microchannels. Estimated wall shear rates demonstrate that experimental conditions reproduce physiologically realistic microvascular flow regimes. Estimated viscosity of base fluids and apparent viscosity are provided. (PDF) [file pone.0327948.s005.pdf]

S5. Viscosity and flow analysis

Volumetric flow rates (Q) were measured using a flow unit sensor (Fluigent, France) for both channel diameters (25 µm and 50 µm) across the pressure range of 20–200 mbar. The complete datasets are provided in Tables S5.1, S5.2, S5.3, and S5.4, corresponding respectively to PBS and plasma suspensions and the mean apparent viscosity.

Base-fluid viscosities at 20 °C are *estimated* from literature manufacturer data using a logarithmic mixing rule: $\eta \approx 2.13$ mPa·s for PBS [2] + OptiPrep (31.5%) [3] and $\eta \approx 2.90$ mPa·s for plasma [4] + OptiPrep (315 µL per mL plasma) [3], with densities for both mixtures near 1.10 g/cm^3 [2,5].

These properties are consistent with *Merlo et al.* (2023), who reported that an iso-osmotic iodixanol buffer ("iodixanol-2"; PBS + 31.5% OptiPrep + 2 g/L BSA) exhibits plasma-like viscosity (∼1.57 mPa·s at 24 °C), density (∼1.10 g/cm^3), and osmolarity (298 mOsm/L), while preserving RBC morphology and deformability and exerting negligible influence on Poiseuille-flow structuration [6].

Table S5.1. Flow rate and mean apparent viscosity measurements for a 25 µm channel with PBS-suspension. Values are in [L/min].

**Pressure [mBar]**	20.0	40.0	60.0	80.0	100.0	120.0	140.0	160.0	180.0	200.0	**Mean app. viscosity [mPa·s]**
**5% Ht**	0.0190	0.0440	0.0700	0.0970	0.124	0.151	0.178	0.203	0.227	0.252	1.97
**10% Ht**	0.0160	0.0410	0.0680	0.0940	0.122	0.149	0.174	0.200	0.227	0.252	1.99
**15% Ht**	0.0190	0.0450	0.0720	0.0990	0.126	0.154	0.181	0.207	0.233	0.259	1.87
**20% Ht**	0.0190	0.0440	0.0700	0.0960	0.123	0.150	0.177	0.202	0.228	0.254	2.02

Table S5.2. Flow rate and mean apparent viscosity measurements for a 25 µm channel with plasma suspension. Values are in [L/min].

**Pressure [mBar]**	20.0	40.0	60.0	80.0	100.0	120.0	140.0	160.0	180.0	200.0	**Mean app. viscosity [mPa·s]**
**5% Ht**	0.0330	0.0580	0.0920	0.128	0.161	0.193	0.224	0.253	0.293	0.325	1.34
**10% Ht**	0.0200	0.0340	0.0560	0.0810	0.109	0.136	0.175	0.212	0.248	0.287	1.94
**15% Ht**	0.0240	0.0560	0.0870	0.123	0.156	0.191	0.224	0.254	0.280	0.308	1.57
**20% Ht**	0.0170	0.0430	0.0800	0.113	0.145	0.178	0.211	0.241	0.269	0.291	1.64

Table S5.3. Flow rate and mean apparent viscosity measurements for a 50 µm channel with PBS-suspension. Values are in [L/min].

**Pressure [mBar]**	20.0	40.0	60.0	80.0	100.0	120.0	140.0	160.0	180.0	200.0	**Mean app. viscosity [mPa·s]**
**5% Ht**	0.373	0.726	1.13	1.54	1.94	2.36	2.76	3.18	3.59	4.00	1.63
**10% Ht**	0.362	0.715	1.07	1.45	1.79	2.18	2.39	2.77	3.23	3.55	1.71
**15% Ht**	0.267	0.632	0.993	1.41	1.77	2.21	2.54	2.60	3.03	3.48	1.96
**20% Ht**	0.308	0.648	1.04	1.44	1.80	2.15	2.59	2.97	3.37	3.77	2.14

Table S5.4. Flow rate and mean apparent viscosity measurements for a 50 µm channel with plasma suspension. Values are in [L/min].

**Pressure [mBar]**	20.0	40.0	60.0	80.0	100.0	120.0	140.0	160.0	180.0	200.0	**Mean app. viscosity [mPa·s]**
**5% Ht**	0.344	0.729	1.18	1.63	2.08	2.48	2.93	3.43	3.82	4.26	1.54
**10% Ht**	0.277	0.733	1.23	1.73	2.04	2.44	2.93	3.21	3.51	3.70	1.86
**15% Ht**	0.333	0.726	1.13	1.64	2.13	2.62	3.10	3.43	3.86	4.30	1.68
**20% Ht**	0.387	0.820	1.29	1.77	2.19	2.59	3.08	3.53	3.90	4.30	1.91

Mean velocity was obtained from the Fluigent flow rate as $U = Q/A = Q/(\pi R^2)$ with $R = D/2$, giving $U \approx 0.5$–$11.0\ \mathrm{mm\,s^{-1}}$ (25 µm) and 2.3–36.5 $\mathrm{mm\,s^{-1}}$ (50 µm).

Reynolds number was computed as $\mathrm{Re} = \rho U D/\mu$ with $\rho = 1060\ \mathrm{kg\,m^{-3}}$ and μ taken as the *mean apparent viscosity* from Tables S5.1–S5.4 (1.34–2.14 mPa · s). This yields Re = 0.006–0.218 for the 25 µm channel and Re = 0.057–1.44 for the 50 µm channel, i.e., fully laminar across conditions.

Wall shear rate followed $\dot{\gamma}_w = 8U/D$, spanning 1.7×10^2–3.5×10^3 s^{-1} (25 µm) and 3.6×10^2–5.8×10^3 s^{-1} (50 µm), consistent with arteriolar/capillary regimes [1].

References

[1] Cho YI, Cho DJ. Hemorheology and microvascular disorders. Korean Circ J. 2011;41(6):287–295. doi: 10.4070/kcj.2011.41.6.287.

[2] Brown PH, Balbo A, Zhao H, Ebel C, Schuck P. Density contrast sedimentation velocity for the determination of protein partial-specific volumes. PLoS One. 2011;6(10):e26221. doi: 10.1371/journal.pone.0026221.

[3] GE Healthcare. Visipaque (iodixanol) injection: prescribing information. Silver Spring (MD): U.S. Food and Drug Administration; 2017 May. p. 31.

[4] Hund SJ, Kameneva MV, Antaki JF. A quasi-mechanistic mathematical representation for blood viscosity. Fluids. 2017;2(1):10. doi: 10.3390/fluids2010010.

[5] Hinghofer-Szalkay H. Volume and density changes of biological fluids with temperature. J Appl Physiol. 1985;59(6):1686–1689. doi: 10.1152/jappl.1985.59.6.1686.

[6] Merlo M, Dupire J, Abkarian M. Influence of storage and buffer composition on the mechanical behavior of flowing red blood cells. Biophys J. 2023;122(4):703–717. doi: 10.1016/j.bpj.2023.01.015.
